# A proposed reverse transcription mechanism for (CAG)n and similar expandable repeats that cause neurological and other diseases

**DOI:** 10.1016/j.heliyon.2020.e03258

**Published:** 2020-02-26

**Authors:** Andrew Franklin, Edward J. Steele, Robyn A. Lindley

**Affiliations:** a Medical Department, Novartis Pharmaceuticals UK Limited, 200 Frimley Business Park, Frimley, Surrey, GU16 7SR, United Kingdom; b Melville Analytics Pty Ltd, Melbourne, Vic, 3004, Australia; c CYO’Connor ERADE Village Foundation, Perth, WA, Australia; d GMDxgenomics, Melbourne, Vic, Australia; e Department of Clinical Pathology, Faculty of Medicine, Dentistry & Health Sciences, University of Melbourne, Vic, Australia

**Keywords:** Neuroscience, CAG expansions, Huntington's disease, Error-prone DNA repair, AID/APOBEC/ADAR deaminases, DNA polymerase-eta, Immunoglobulin somatic hypermutation

## Abstract

The mechanism of (CAG)n repeat generation, and related expandable repeat diseases in non-dividing cells, is currently understood in terms of a DNA template-based DNA repair synthesis process involving hairpin stabilized slippage, local error-prone repair via MutSβ (MSH2–MSH3) hairpin protective stabilization, then nascent strand extension by DNA polymerases-β and -δ. We advance a very similar slipped hairpin-stabilized model involving MSH2–MSH3 with two key differences: the copying template may also be the nascent pre-mRNA with the repair pathway being mediated by the Y-family error-prone enzymes DNA polymerase-η and DNA polymerase-κ acting as reverse transcriptases. We argue that both DNA-based and RNA-based mechanisms could well be activated in affected non-dividing brain cells *in vivo*. Here, we compare the advantages of the RNA/RT-based model proposed by us as an adjunct to previously proposed models. In brief, our model depends upon dysregulated innate and adaptive immunity cascades involving AID/APOBEC and ADAR deaminases that are known to be involved in normal locus-specific immunoglobulin somatic hypermutation, cancer progression and somatic mutations at many off-target non-immunoglobulin sites across the genome: we explain how these processes could also play an active role in repeat expansion diseases at RNA polymerase II-transcribed genes.

## 1 Purpose of this article

It is necessary to directly state our overarching philosophy and rationale up front. We are molecular and cellular immunologists interested in the reverse transcriptase (RT) mechanism of antigen-driven somatic hypermutation (SHM) of rearranged immunoglobulin (Ig) variable region genes (Franklin et al., 2004; Steele et al., 2004; Steele et al., 2006; Steele, 2009; Steele, 2016; Steele and Lindley, 2017, Steele, 2017; these papers should be consulted for the molecular details of the RT Ig SHM mechanism). Why should we be applying mechanisms implicated in Ig SHM to the molecular events that precipitate (CAG)n and related trinucleotide repeat (TNR) disease? Somatic hypermutation underpins the generation of diversity in adaptive immunity in response to antigenic challenge, and while this response has physiologic benefit, it can also have pathologic consequence (such as in the case of cancer progression). In our view the generation of (CAG)n and related TNR may be interpreted as representing as a dysfunctional ‘Ig-SHM-like’ response allowing the postulate that the same or similar molecular processes might be involved. Viewing the molecular generation of trinucleotide repeat diseases through this prism provides fresh perspective and we hope contributes to advancement of the field.

**Figure 1:**
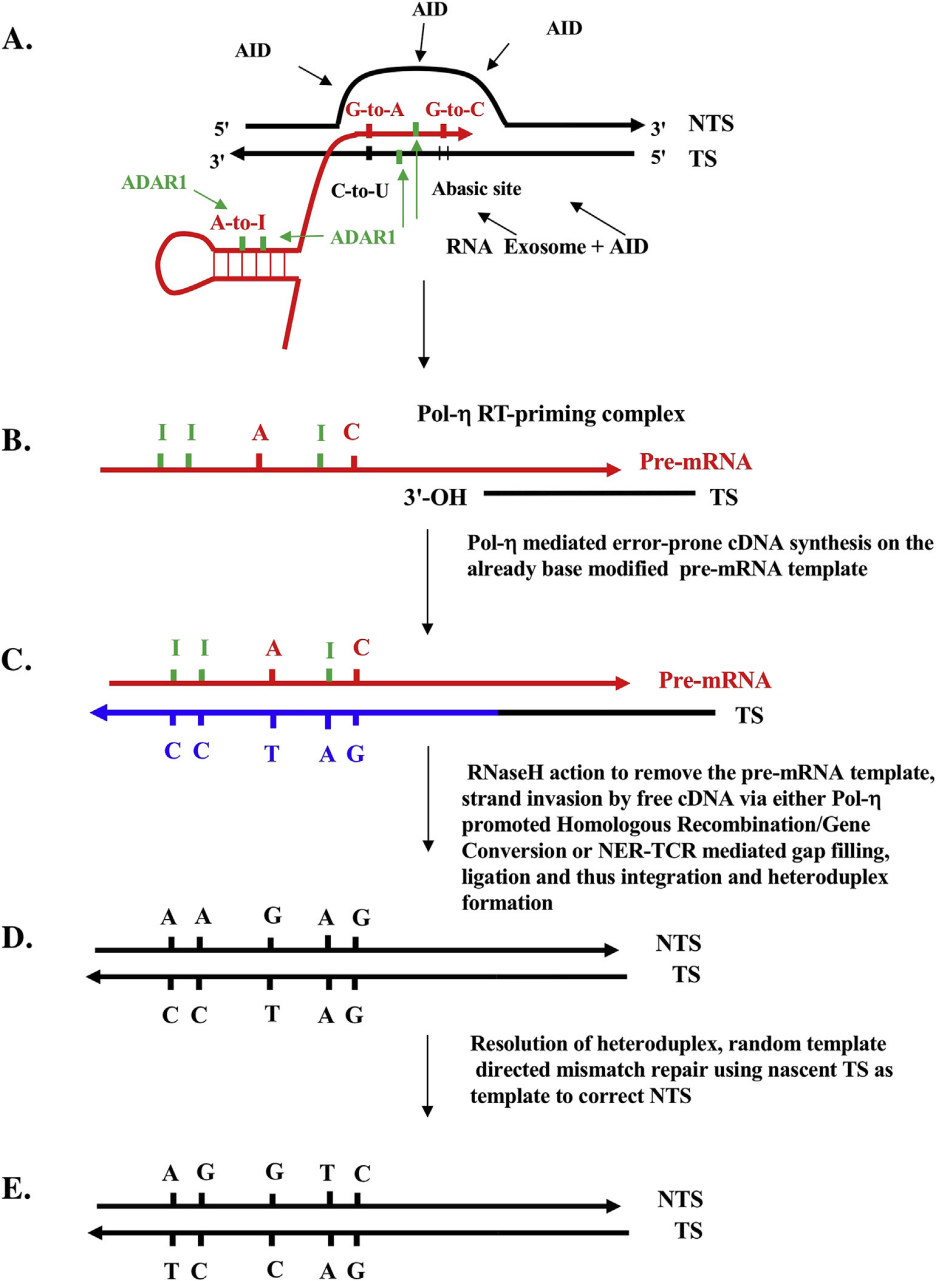
Reverse transcriptase mechanism of immunoglobulin somatic hypermutation. Adapted from previous papers and as discussed in Box 1 and in the text (Franklin et al., 2004; Steele et al., 2006; Steele 2009, 2016; Steele and Lindley 2010, 2017; Lindley and Steele, 2013). Primary references can be found in these papers. The mechanism is an adaptation of the target site reverse transcriptase (TSRT) process first described in Luan et al. (1993). Independent confirmation (Box 2) that human DNA polymerase-η is a reverse transcriptase (RT) has been published recently by Su et al. (2017, 2019). The steps are: A. Modifications of DNA and pre-mRNA sequences at transcription bubbles via C-to-U and A-to-I deamination events, RNA polymerase II misincorporations (Kuraoka et al., 2003). into the pre-mRNA; B. DNA polymerase-η RT-priming complex; C. cDNA synthesis; D. cDNA strand invasion and heteroduplex formation; E. Resolution of the heteroduplex prior to DNA replication and cell division (MacPhee, 1995). Shown in A is the role of the RNA exosome at transcription bubbles in allowing access of AID to unpaired C-sites in the RNA:DNA hybrid (Basu et al., 2011). The key C-to-U deamination step at CAG repeats, from A. to B., is expanded in Figure 2. See Box 1 for further explanation. Black lines, DNA. Red lines RNA. Blue lines, cDNA. NTS, non-transcribed strand. TS, transcribed strand.

**Figure 2:**
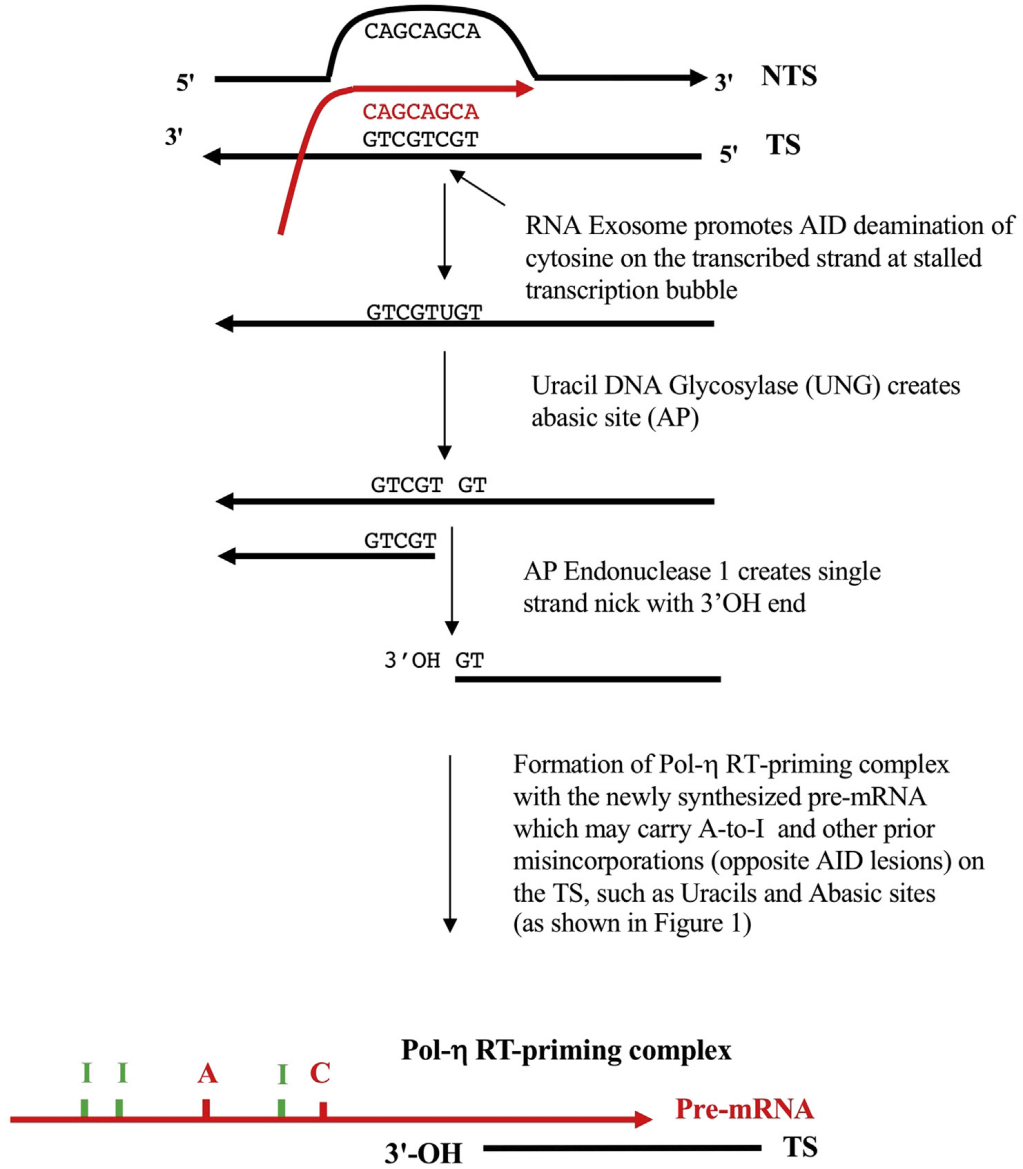
Formation of DNA polymerase-η reverse (Pol-η) reverse transcriptase (RT)-priming sites at activation-induced deaminase (AID) lesions on the transcribed stand (TS) at stalled transcription bubbles initiating target site reverse transcription (TSRT). A CAG repeat within a transcription bubble is shown with tandem WGCW motifs that allow the hypothesized AID targeted deamination events. This illustrates the key AID-mediated C-to-U deamination lesion, and somatic hypermutation (SHM)-initiating step discussed in the text and in step A. in Figure 1, leading to the formation of the Pol-η RT priming step (step B. in Figure 1). This step is well documented in the immunoglobulin (Ig) SHM literature, see references in Franklin et al., (2004) and other reviews (Di Noia and Neuberger, 2007; Teng and Papavasiliou, 2007; Maul and Gearhart, 2010; Steele, 2016). A recent paper by the Gearhart group demonstrates further the generation of such single stranded nicks with 3′-OH ends (Zanotti et al., 2019). The focus here, and in Figure 1, is on how the TS is deaminated as it is *this TS lesion* which sets up the sequelae of downstream steps (Figure 1) that lead to the key *diagnostic* strand biases in Ig SHM data sets discussed in the text here, namely mutations at A sites exceed mutations at T sites (A >> T) and, *in the same data sets*, mutations at G sites exceed mutations at C sites (G >> C). These A-site mutations are triggered primarily by the ADAR1 mediated A-to-I modifications in the nascent pre-mRNA (Steele et al., 2006). Both these counter intuitive strand biases at A:T and G:C base pairs occur in all Ig SHM data sets (Steele, 2009) and in all off-target non-Ig data sets analysed (e.g. TP53 substrates, in numerous different cancers, Lindley and Steele, 2013). They cannot be explained by utilization of the alternative templates for DNA repair synthesis, namely the non-transcribed strand (NTS) as copying templates, thus the focus here on the TS rather than the NTS. These alternatives, particularly in relation to Pol-η-mediated DNA repair, are discussed in detail in Steele et al., (2006) and Steele (2016). It is certainly accepted that alternative explanations exist for transcription coupled repair (TCR) strand-biases in somatic mutations generated by bulky adducts (Denissenko et al., 1996, 1998), which lead to Nucleotide Excision Repair (NER)-TCR and preferential repair of the TS as discussed in detail in Figure 4. For the minor base alterations considered here, simple copying errors at AID lesions by RNA polymerase II misincorporation come into play (Kuraoka et al., 2003); certainly low level 8oxoG generated lesions by reactive oxygen species (ROS) do not result in NER-TCR strand biases on DNA repair (Thorslund et al., 2002). This may not be the case for significant clusters of 8oxoG lesions at repetitive WG sites as would be the case for ROS lesions at (CAG)n tracts in expressed genes in brain cells (Figure 4 legend and references). Thus, the AID deamination of cytosines on the TS assisted by the RNA exosome (Basu et al., 2011) leads as shown to uracil in the TS which is usually removed by a uracil DNA glycosylase (i.e. UNG) which then set up the substrate for cleavage of the abasic site by an apurinic/apyrimidinic endonuclease (i.e. APE1) and generation of free 3′OH termini on the TS. These 3′OH ends, on annealing to the newly synthesised pre-mRNA at this transcribed genomic site, create the hypothesised Pol-η RT-priming complex as shown (and step B in Figure 1).

Our approach thus differs from other investigators working within the traditional discipline of (CAG)n and related TNR expansion diseases. In coding regions, TNR expansions occur in-frame, thus naturally lending themselves to an alternative RT-based explanation for their genesis. This is the purpose of this “Hypothesis” article and review. We are not supplanting the existing molecular mechanisms but rather adding to them by providing plausible and testable explanations for TNR expansions in RNA Pol II-transcribed regions arising from plausible RT processes transposed from our understanding of the molecular immunology of Ig SHM phenomena which may involve DNA synthesis opposite the pre-mRNA template of the target gene (Figures 1 and 2). We can say this now because we have shown that C-site and A-site off-target ‘Ig SHM-like’ mutagenic responses mediated by DNA and RNA deaminases (adenosine deaminases acting on RNA [ADARs], activation-induced deaminase [AID] and apolipoprotein B mRNA editing catalytic polypeptide-like proteins or APOBECs) appear to be operative also across the genome during cancer progression (Steele and Lindley 2010, 2017; Lindley, 2013; Lindley and Steele, 2013; Lindley et al., 2016; Mamrot et al., 2019). In our view, an understanding of these non-Ig off-target ‘Ig SHM-like’ mutagenic responses is related to our proposal here. That is, a molecular mechanism of TNR expansions may well involve pre-mRNA intermediates, as has been implicated to explain the molecular mechanism of Ig SHM in adaptive immunity and Ig SHM-like mechanisms in cancer progression.

## 2 TNR expansion diseases

**Figure 3:**
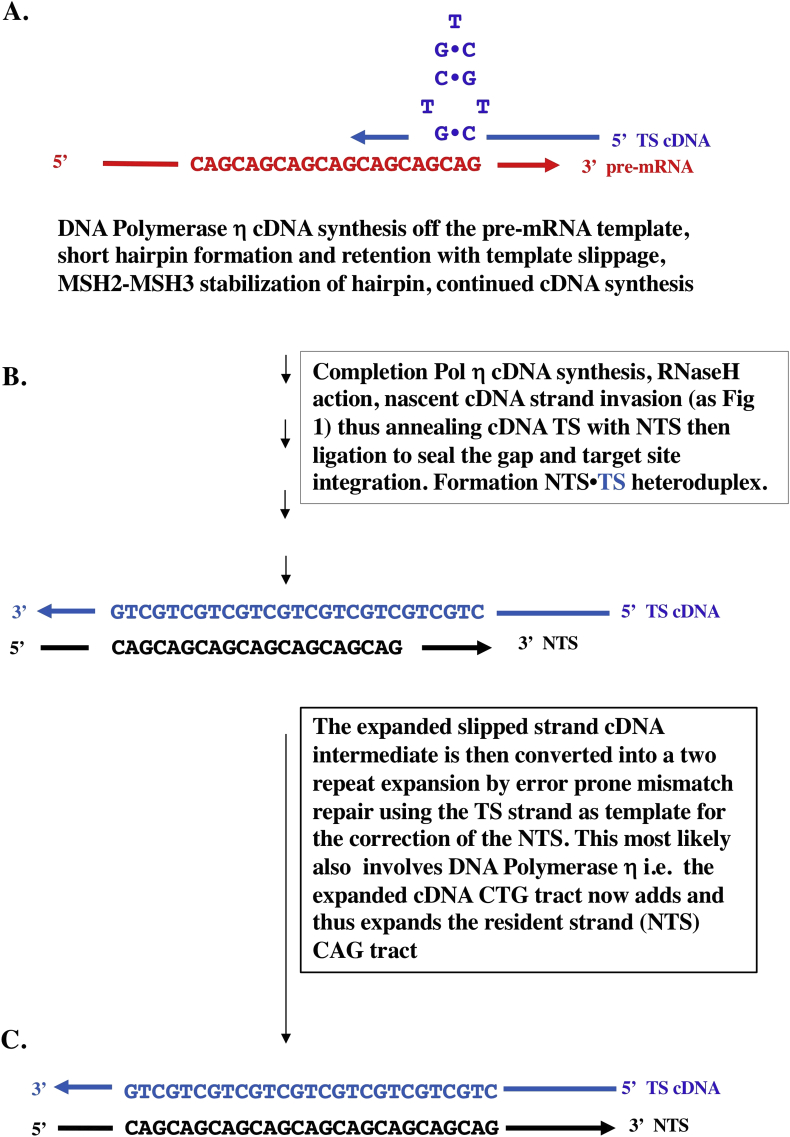
Reverse transcriptase mechanism of CAG repeat expansion. General schematic mechanism adapted in part from prior published papers (Panigrahi et al., 2005; Mirkin, 2007; Chan et al., 2013; Guo et al., 2016), but now using a pre-mRNA template. A. Hairpin formation (stabilized by MSH2–MSH3) and template slippage of cDNA during reverse transcription opposite pre-mRNA B. Strand invasion of duplex DNA by cDNA which anneals with the NTS to replace the TS. C. Resolution of the heteroduplex by mismatch repair. Repeat contractions could occur via endonuclease removal of the hairpin prior to reverse transcription. Note that all the post-reverse transcription steps specified in Figure 1 apply here. Thus, ‘strand invasion’ as addressed in the text and in Box 1, and in Figure 1 legend, can involve either short (∼30 nt) or long (>100 nt) cDNA tracts. Strand invasion could occur via either the homologous recombination/gene conversion promoting properties of DNA polymerase-η (Kawamoto et al., 2005; McIlwraith et al., 2005) or a reverse transcription-driven RNA-templated NER-TCR process for TCR gap tracts ∼30 nt (discussed in the text, Box 1, in Figure 1 legend, and outlined in detail in Figure 4). CAG, cytosine-adenine-guanine; cDNA, complementary DNA; MSH2, MutS homologue 2; MSH3, MutS homologue 3; NER, nucleotide excision repair; nt, nucleotide; NTS, non-transcribed strand; TCR, transcription-coupled repair; TS, transcribed strand.

**Figure 4:**
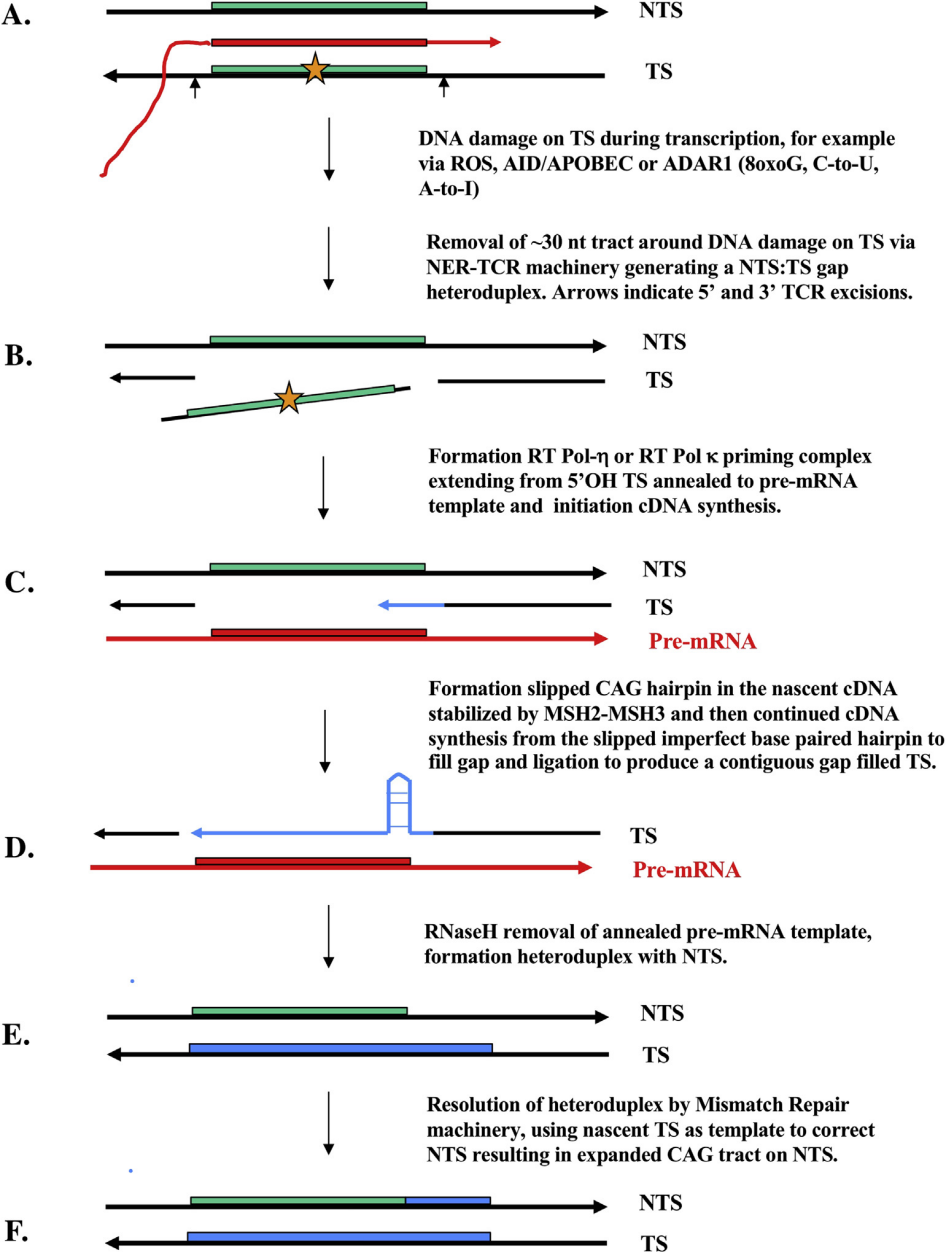
The reverse transcription mechanism for CAG expansions which invokes the alternative DNA polymerase-η-mediated (or DNA polymerase-κ-mediated) RNA-dependent DNA synthesis mechanism coupled to the nucleotide excision repair–transcription-coupled repair (NER-TCR) process for short TCR gap tracts of ∼ 30 nucleotides (nt). Green, red, blue rectangles are CAG repeat tracts. Other lines are DNA in black, cDNA in blue, and pre-mRNA in red. **A**. A transcribed region over a (CAG)n tract. This is a schematic and not meant to show a 9–10 nt RNA:DNA hybrid at a transcription bubble (as in Figures 1 and 2), but to convey the idea of transcription across the tract. The gold star indicates significant DNA damage(s) to the transcribed strand (TS), and thus sensed by the RNA polymerase II elongation complex, which could be due to clustered mutagenic episodes of dysregulated enzyme-mediated nucleic acid deamination by AID/APOBEC and ADAR at appropriate tandem C-site and A-site deamination motifs exposed at the RNA:DNA hybrid, on the TS itself and on the displaced non-transcribed strand (NTS) (Basu et al., 2011; Zheng et al., 2017) as discussed in Steele and Lindley (2017) or conceivably of reactive oxygen species (ROS) modifying guanines at tandem WG sites creating a significant lesion on the TS (clustered 8oxoG lesions, common at CAG repeat tracts as described in Polyzos and McMurray, 2017, and see the SBS18 WG signature in https://cancer.sanger.ac.uk/cosmic/signatures). Steps A through F summarize the potential events hypothesized (also see Box 2). Following sensing of damage on the TS, the RNA polymerase II elongation complex stalls, backtracks and allows the NER machinery of the TCR supramolecular complex (Hanawalt and Spivak, 2008; Spivak, 2016) to make 5′ and 3′ excisions in the TS around the lesion (A) and thus release the damaged TS region (B), exposing the single-stranded gapped section of the NTS. Unlike normal TCR where the NTS would be the template for the replicative DNA polymerases-δ or -ε, the proposed model here (C) invokes co-option of the pre-mRNA (normal sequence or possibly also base modified, as shown in Figure 1) as the template for gap repair reverse transcription by DNA polymerase-η or even DNA polymerase-κ (Box 2), given that the latter is known to repair such TCR gaps (Ogi and Lehmann, 2006). Slipped MSH2–MSH3-stabilized CAG repeat hairpins (as in Figure 3) form on the pre-mRNA template and complementary DNA (cDNA) synthesis continues to fill the gap (D). After RNase H activity, the reformation of the TS:NTS heteroduplex (E) then sets the stage for resolution of the nucleotide differences using the TS as the template for correction of the NTS (F). ADAR, adenosine deaminase acting on RNA; AID, activation-induced deaminase; APOBEC, apolipoprotein B mRNA editing catalytic polypeptide-like; MSH2, MutS homologue 2; MSH3, MutS homologue 3.

The current understanding of the molecular mechanism responsible for the expansion of DNA repeat sequences is incomplete (Polyzos and McMurray, 2017). Thus we are advancing our model as a potential additional general mechanism for expandable TNR repeats in the main TNR expansion diseases in non-dividing cells in the brain – especially, those TNR expansion diseases involving TNRs that can form imperfect yet stabilized snap-back Watson-Crick base-paired hairpin structures on the pre-mRNA template (see Figure 3). So, in addition to (CAG)n, this would also apply particularly to (CTG)n and (CGG)n expansion diseases. It may also apply to the long GAA tract expansions of Friedreich's ataxia (FRDA), because more conventional DNA slippage could occur during replication involving the far weaker Hoogsteen (non-Watson-Crick) base pairing that would help create stabilized hairpins/R-loops (as listed in Table 1 of Usdin et al., 2015). So the model advanced in Figures 3 and 4 is most directly applicable, in terms of Watson-Crick base pairing, to (CAG)n codon expansions, but it could also apply to expansions affecting untranslated regions (UTR), such as 3′-UTR (CTG)n and 5′-UTR (CGG)n expansion diseases, again also involving slippage during replication (see Table 1 in Usdin et al., 2015). Since our model applies to non-dividing cells in the brain, the emphasis here is on AID/APOBEC and ADAR deaminations coupled with transcription-linked DNA processes like target site reverse transcription rather than DNA replication.

In the brain and central nervous system, there are numerous hereditary TNR expansion diseases and their somatic progression correlates (Mirkin, 2007; Usdin et al., 2015). They are often referred to as “Dynamic Mutations” (Richards, 2016) to distinguish them from more conventional and stable forms of Mendelian mutations. These diseases occur and somatically progress in severity in affected families with an earlier age of onset over successive generations. Dynamic mutations of the repeat sequence type in protein-coding regions are thus associated with molecular mechanisms responsible for non-Mendelian genetic inheritance phenomena often thought of as ‘anticipatory’. We now know from data generated using mouse models of Huntington's disease that suppression of somatic expansion delays the onset of associated pathophysiology, suggesting that it is not so much CAG copy number but perfect CAG repeat copy number that rate limits somatic expansion and pathology in Huntington's disease (Budworth et al., 2015). Nevertheless, excessively expanded repeats result in fragile sites on chromosomes (Richards, 2016) and thus have genomic neurodegenerative disease consequences in the non-dividing cells of the brain, producing aberrantly misfolded proteins and toxic RNA transcripts (Mirkin, 2007; Nalavade et al., 2013; Usdin et al., 2015).

Perhaps best known are the (CAG)n or polyQ expansion diseases such as spinal bulbar muscular atrophy (SBMA), dentatorubral pallidoluysian atrophy (DRPLA), Huntington's disease (HD), and various variants of spinocerebellar ataxia (SCA1,2,3,6,7,12,17). Other diseases include: the (CTG)n expansions such as myotonic dystrophy type 1 (DM1), Huntington's disease-like 2 (HDL2) and spinocerebellar ataxia 8 (SCA8); the (CGG)n expansion diseases such as fragile X syndrome (FXS) and related syndromes, and the (GAA)n expansion diseases such as Friedreich's ataxia (FRDA). Very good and generally agreed upon summaries of the various genes that can be affected, their normal repeat lengths and the range and extent of pathogenic repeat lengths can be found in Table 1 of Usdin et al. (2015), Table 2 of Richards (2016), and also in Figure 1 of Polyzos and McMurray (2017). The current expansion mechanisms invoke MutS homologue (MSH)2–MSH3 hairpin-stabilized initiation during replication on the leading strand (Mirkin, 2007) or, the now consensus view, more generally during local DNA repair across the genome (Polyzos and McMurray, 2017). That is, as we discuss later, local error-prone DNA repair on the nascent gap-filling strand assisted via MSH2–MSH3 hairpin-stabilized configurations aiding priming initiation (Panigrahi et al., 2005; Mirkin, 2007; Polyzos and McMurray, 2017), and possibly involving DNA polymerases-β and -δ (Chan et al., 2013; Guo et al., 2016).

The first molecular description of (CAG)n expansion tracts was by La Spada et al. (1991) in mutations causing X-linked SBMA of the androgen receptor gene. This initial study clearly showed that in diseased individuals, the CAG repeat tracts expand in the 5′-to-3′ direction (i.e. with the same transcriptional polarity of the pre-mRNA). This was soon followed by a similar study showing CAG expansion tracts in exon 1 of the IT15 gene which encodes the huntingtin protein in HD patients (MacDonald et al., 1993). We now know that about 30 or more hereditary diseases in humans (as curated at the OMIM database) result from similar somatic expansions of codon and other longer repeats in intronic or UTR encoding genomic DNA (Mirkin, 2007; Nalavade et al., 2013; Usdin et al., 2015; Richards, 2016; Polyzos and McMurray, 2017). Such expanded DNA in transcribed regions results in physiologically ‘toxic’ structural features in both aberrantly misfolded proteins and mRNA transcripts, and are thus disruptive to basic biochemical pathways. As discussed, the repeat expansions also occur in the transcribed non-protein coding 5′ and 3′ flanks and intronic regions causing debilitating diseases also through abnormal folding of proteins and RNA transcripts (Mirkin, 2007; Nalavade et al., 2013; Usdin et al., 2015).

## 3 Function of polyQ and related TNR homopolymer tracts in health and disease

PolyQ and similar repeat codon tracts occur in most proteins (Oma et al., 2004; Willadsen et al., 2013), presumably assisting normal interaction with other proteins, intracellular localisation and supramolecular complexes (Oma et al., 2004; Willadsen et al., 2013; Huttlin et al., 2017). In healthy individuals, the lengths for the huntingtin gene (CAG)n repeats range from n ∼ 10–30, yet when they expand to n >37 repeats, overt and progressive HD symptoms become apparent (La Spada et al., 1991; MacDonald et al., 1993; Mirkin, 2007; Nalavade et al., 2013; Usdin et al., 2015; Richards, 2016; Polyzos and McMurray, 2017).

Thus, homopolymer tracts are a normal feature of most proteins. Across representative animal and plant species examined, many normal (wild-type) cytoplasmic proteins have functional polyQ tracts (Willadsen et al., 2013). An analysis of six different species including *Saccharomyces cerevisiae, Arabidopsis thaliana, Caenorhabditis elegans, Drosophila melanogaster*, *Mus musculus* and *Homo sapiens* identified the number of TNR tracts in proteins as 247, 1,947, 559, 3,996, 79,727 and 35,736, respectively (Willadsen et al., 2013). This implies that the great majority of protein-coding genes have embedded TNR tracts serving interactome network functions (Huttlin et al., 2017) involving ‘supramolecular protein-nucleic acid complexes’ such as transcription factor ‘mosaic’ assemblies in promoter regions, as well as complex molecular machines such as ribosomes, spliceosomes, RNA polymerase II elongation complexes and the DNA replication apparatus.

PolyQ and similar amino acid repeat tracts also support a range of protein-protein interactions necessary for the dynamic processing of autologous proteins. For example, a protein widely expressed in the brain is ataxin, a deubiquitinating enzyme. Its polyQ domain in wild-type ataxin-3 enables it to interact with the key autophagy initiator beclin 1. This involves an interaction that sanctions the protection of beclin 1 from proteosome-mediated degradation and thus normal progression into autophagy pathways (Ashkenazi et al., 2017).

## 4 Dysregulated mutagenic activity of deaminases and base sequence features of (CAG)n and similar tracts

AID/APOBEC-mediated C-to-U are predicted to target C residues that are in the primary repeat motif WGCW in HD and related diseases (W = A or T; targeted C residue underlined). Thus, in relation to known mutagenic targeting preferences of AID/APOBEC and ADAR deaminases across diseased genomes (e.g Lindley, 2013; Lindley et al., 2016), (CAG)n and related repeats such as (CTG)n, (CGG)n, (GAA)n, as well as longer repeats such as (CCTG)n and (ATTCT)n (see Table 1 in Usdin et al., 2015) possess features that could lead to dysregulated and thus pathogenic deaminase targeting.

Ig variable region exons are assembled during B cell development from variable (V), diversity (D) and joining (J) gene segments and are hence referred to generically as V(D)J sequences. WGCW motifs are enriched at V(D)J sequences and exist as tandemly arranged repeats (Di Noia and Neuberger, 2007; Teng and Papavasiliou, 2007; Steele, 2016). These tandemly arranged C-centered motifs are targeted by AID in a regulated manner at transcription bubbles at rearranged V(D)J genes to initiate Ig SHM in B cells (Maul and Gearhart, 2010; Heltzel and Gearhart, 2019). They are also enriched at switch regions as part of the transcription-dependent R-loop formation during Ig class switch recombination (Yu and Lieber, 2003; Maul and Gearhart, 2010; Heltzel and Gearhart, 2019). Thus, during antigen-stimulated Ig SHM episodes at V(D)J loci in germinal center B cells *in vivo*, multiple nearby WGCW motifs, often overlapping and concentrated in the sequences termed complementarity determining regions which encode epitope contact residues, can be C-to-U-deaminated by AID causing staggered nicks (after base excision repair generating abasic sites and APE1 endonuclease action, Figure 2) and potentially double-strand breaks (DSBs). These DSBs are ideal targets for DNA repair via homologous recombination (Jasin and Rothstein, 2013) and gene conversion processes, both of which are able to be stimulated by DNA polymerase-η (McIlwraith et al., 2005; Kawamoto et al., 2005, Box 1). Such DSB lesions can also be the target of RNA-templated DNA repair in yeast model systems mediated presumably by replicative DNA polymerase-α and -δ (Storici et al., 2007, Box 2). This is an important point in regard to the mechanism shown in Figure 1 as DNA polymerase-η is the sole error-prone DNA polymerase known to be involved in physiological Ig SHM *in vivo* (Delbos et al., 2007). More information around the RT activity of human DNA polymerase-η is outlined in Box 2.

Box 1 **How does the cDNA invade to form a heteroduplex?**

This explanation relates to steps B–E in Figure 1. How does the cDNA corresponding to the TS invade to form the first heteroduplex with the NTS (steps C and D in Figure 1)? Step B shows the predicted, and necessary, DNA polymerase-η RT-priming complex of nicked TS DNA with a 3′OH end annealed to the pre-mRNA. In previous iterations of the RT model (Franklin et al., 2004), the strand invasion step has been assumed to occur once the target site reverse transcription step is initiated as in Luan et al. (1993). The cDNA is initiated and is contiguous with the TS on the 5′ side.

There are at least two types of ‘strand invasion’ mechanisms following RNaseH removal of the pre-mRNA from the RNA:cDNA heteroduplex (Step C). For long cDNA tracts (>100 nt) we assume that the known homologous recombination/gene conversion promoting properties of human DNA polymerase-η allow this to happen (again after RNase H removes the annealed template pre-mRNA), as in the canonical immunoglobulin variable region gene conversion system in chicken B cells (Kawamoto et al., 2005; McIlwraith et al., 2005). For shorter tracts of up to 30 nt, which is the approximate normal maximum tract length of most CAG repeats in the known genes subject to repeat expansion (Usdin et al., 2015; Richards, 2016), it is expected that the NER-TCR machinery will act to generate excisions on both the 5′ and 3′ side of the damaged TS around the stalled transcription bubble, exposing an ∼ 30 nt gap (Bowman et al., 1997). Normal TCR-directed gap filling (see Fig. 2 in Hanawalt and Spivak, 2008; Spivak, 2016) is now templated for cDNA synthesis of the TS opposite the pre-mRNA as shown in steps B and C, followed by ligation on the 3′ side of the gap as heteroduplex is formed (Step D). In further support of the TCR alternative for ‘strand invasion’ is the fact that the gap filling to re-synthesize the ∼30 nt TS gap, which equates to the excised section in normal TCR damage responses (Hanawalt and Spivak, 2008; Spivak, 2016), is a step that involves DNA polymerases-δ, -ε and/or Y-family DNA polymerase-κ (see Ogi and Lehmann, 2006). This is important in relation to the RT model for somatic hypermutation outlined in Figure 1 (and for potential CAG expansions as shown in Figure 3) because three of the human Y-family polymerases (i.e. DNA polymerases-η, -ι and -κ) can perform RNA-dependent DNA synthesis, as previously shown by Franklin et al. (2004) and see Box 2. This now adds to the likelihood that a RT-driven RNA-templated NER-TCR process may happen in normal cases of translesion bypass repair. Indeed RNA-templated DNA repair of double strand breaks has also been demonstrated in *S. cerevisiae* by Storici et al., (2007) (e.g. involving yeast replicative DNA polymerases-α and -δ; see comparable primer extension data of ≥ 5–10 nt opposite RNA templates in Fig. 3 in that paper).

In summary, the cDNA strand invasion steps for C through D in Figure 1 can, from available evidence, be executed for long cDNA tracts by the established homologous recombination/gene conversion promoting properties of human DNA polymerase-η (for tracts >100 nt), or via a DNA polymerase-η or DNA polymerase-κ RT-driven RNA-templated NER-TCR process for TCR gap tracts of ∼30 nt. The potential steps of the second alternative for CAG expansions are outlined in Figure 3 in some detail. cDNA, complementary DNA; NER, nucleotide excision repair; nt, nucleotide; NTS, non-transcribed strand; TS, transcribed strand; RT, reverse transcriptase; TCR, transcription-coupled repair.

Alt-text: Box 1

Box 2 **Reverse transcriptase activity of DNA Polymerase-η and DNA Polymerase-κ**

During the peer review of this article, the basic validity of the reverse transcriptase (RT) model of immunoglobulin somatic hypermutation driven by the RNA-dependent DNA synthesis activity of human DNA polymerase-η was queried, mainly because of our inappropriate citation of key papers by the Su et al. group. We should have cited both Su et al., (2017) and Su et al., (2019) rather than just the more recent 2019 paper of the group. This confusion nevertheless generated this useful clarifying Glossary Box which now addresses this sceptical viewpoint which we believe is reasonably widespread in the molecular immunology community (at least). The *in vitro* observations in Franklin et al. (2004) on human Y-family DNA translesion DNA polymerases-η, -κ, -ι were made using an indirect, PCR-based detection method, a product enhanced real time (PERT) PCR assay. Moreover, although Su et al. (2017, 2019) recently demonstrated independently the RT activity of human DNA polymerase-η, the efficiency of polymerization that was reported in the Su et al., (2019) paper, in contrast to that in Su et al., (2017), was very low opposite RNA template relative to DNA template, with no primer extension being observed beyond incorporation of only a single nucleotide. So just reading Su et al., (2019) by itself can create the misleading impression that human DNA polymerase-η is a poor cellular RT that is hardly likely to support the continuous insertion of dozens of nucleotides as we have proposed in this paper (and see Blanden et al., 2004).

So we have now critically re-read the key papers on this issue by Su et al. (2017, 2019), particularly the primer extension assays used in the earlier article (Su et al., 2017). These workers annealed short oligonucleotide sequences *in vitro* to create DNA/RNA substrates. The primer extension data in these *in vitro* biochemical primer extension assays with purified enzymes completely confirm our prior work and conclusions using the PERT assay (Franklin et al., 2004). Our work showed that elongation of complementary DNA (cDNA) copies opposite the MS2 phage RNA template annealed to a DNA primer yielded extension products of at least 27–37 nucleotides. The critical confirmatory data is presented in Figure 4 in the report by Su et al. (2017), which shows DNA primer extension data opposite RNA template. Specifically, a 5′-to-3′ DNA primer was annealed to a longer 3′-to-5′ RNA template (longer by 5 nucleotides); the maximum possible product size generated by cDNA extension of the DNA primer that could be detectable by polyacrylamide gel analysis was 5 nucleotides.

**Figure:**
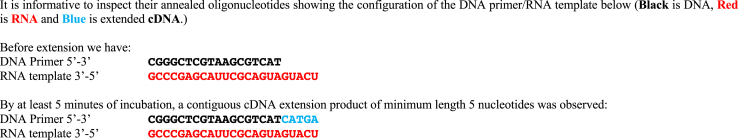

These data compare favourably with the minimum 27–37 nucleotide cDNA products within 1 h incubation detected in the PERT assay of Franklin et al. (2004). So the primer extension data of Su et al., 2017 provide a clear confirmatory demonstration of genuine reverse transcription not only by human DNA polymerase-η but also to a lesser extent by human DNA polymerase-κ for purified enzymes *in vitro* as in Franklin et al. (2004). The relative RT efficiencies reported are also similar to those reported in Franklin et al. (2004). Thus the comparison with the more efficient HIV-1 RT is very informative and also similar to the relative comparisons in Franklin et al. (2004). But the additional enzyme kinetic information reported in Su et al., (2017) (in their Table 1) is new quantitative data and very important in the understanding of the relative efficiency of the RT activity of human DNA polymerase-η. The insertion of dC opposite template rG (as a measure of catalytic efficiency) for both human DNA polymerase-η and HIV-1 RT is *very similar*, allowing them to conclude that human DNA polymerase-η is a relatively efficient cellular RT. While similar results in principle to human DNA polymerase-η were observed for human DNA polymerase-κ, the latter polymerase was clearly less efficient as compared to the former in terms of RNA-dependent DNA synthesis activity (Su et al., 2017).

It is important to qualify that all of these biochemical data were gathered under conditions *in vitro* using purified polymerases. These conditions are clearly far removed from *in vivo* physiological conditions in living cells which in most well studied cases involve supramolecular complexes and regulated interactions of many functional proteins among themselves and with nucleic acid molecules. Thus, *in vivo*, we should expect the replication clamp proliferating cell nuclear antigen (PCNA) with the single strand stabilizing proteins replication protein A (RPA) with replication factor C (RFC) to participate in improving and regulating the processivity of the DNA polymerases, particularly translesion human DNA polymerase-η (Haracska et al., 2001). Indeed we conducted *in vitro* experiments involving PCNA, RPA and RFC and found that human DNA polymerase-η activity in the PERT assay was enhanced at least four-fold by the addition of these proteins (Franklin, 2004), a result which implies that under *in vivo* conditions in living cells, processive cDNA synthesis can be expected. Long tract cDNA synthesis *in vivo* via the RT activity of human DNA polymerase-η is expected at rearranged immunoglobulin loci (Blanden et al., 2004) and is expected when the 5’ boundaries of the distribution of somatic mutations are critically evaluated (Blanden et al., 2004). We note that Krijger et al. (2011) have shown ubiquitination-independent PCNA activation of DNA polymerase-η during physiological *in vivo* somatic hypermutation and DNA damage tolerance in a murine system.

The follow up work reported in Su et al. (2019) uses human cell extracts. In these experiments (see Figure 4 in Su et al., 2019), the “presence of RNA strands in the annealed DNA primer/RNA template complex caused the substrates to be degraded more easily than with the DNA/DNA substrate.” They speculate that this extensive degradation is probably caused by RNase H1 and RNase H2 in the cell extracts. So it is actually a race in these experiments to extend the cDNA product by 5 nucleotides *before degradation* of the substrate complex (or extension products themselves). Nevertheless, the authors report clear human DNA polymerase-η-dependent cDNA extensions of one nucleotide opposite the RNA template, which allows the authors to conclude that in these ‘cellular environments’ the results demonstrate the critical role of human DNA polymerase-η in reverse transcription and indicate that human DNA polymerase-η is a key reverse transcriptase in human cells (Su et al., 2019), thus extending their work on purified polymerases in primer extension assays *in vitro* (Su et al., 2017).

Alt-text: Box 2

The tandem CAG, CTG and CGG repeats in coding regions or UTRs in many protein-coding genes (targeted C residues underlined) will thus provide rich targets for cytosine deamination if the AID/APOBEC deamination events become dysregulated and go ‘off target’ as appears to occur across the genome in cancer (Lindley, 2013; Lindley and Steele, 2013; Lindley et al., 2016). Thus, in this context, the polyP-encoding tract in exon 1 of the Huntington's gene (IT15), just downstream of the polyQ-encoding tract, also provides an ideal deamination substrate for other cytidine deaminases known to have target substrates that overlap those of AID. For example, CCN motifs accessible in single-stranded DNA (ssDNA) substrates, such as occurs during displacement of the non-transcribed strand (NTS) at translocating transcription bubbles (Figure 1), could serve as substrates for editing by APOBEC3G (Beale et al., 2004). In this regard the reverse complement of the common 5′UTR repeat nucleotide triplet CGG in FXS and related syndromes (i.e. CCG) is a common APOBEC3G motif and would represent a potential tandem array deamination target in the DNA of an expressed gene.

In the same vein, the intronic (ATTCT)n repeat (as seen in spinocerebellar ataxia 10 [SCA10]) and the (GAA)n expansion intronic repeats (as seen in FRDA) present as possible transcription-linked ADAR1 motifs in both nascent double-stranded (dsRNA) hairpins and RNA:DNA hybrids (Zheng et al., 2017; Steele and Lindley, 2017). The RNA:DNA hybrid target substrates are similar to the numbers of WA-rich targets both within the intronic Alu repeat elements themselves (the well known WA rich sequences in the Alu central region) and their inverted, and spaced, Alu snap-back derivatives, that are known to constitute the main targets of ADAR-mediated A-to-I RNA editing *in vivo* in the brain (Paz-Yaacov et al., 2010; Picardi et al., 2015).

Returning to the cancer analogy and the link with deaminase action, as with the triggering of Ig SHM itself (reviewed in Di Noia and Neuberger, 2007, Teng and Papavasiliou, 2007, and again in Steele, 2009, Maul and Gearhart, 2010, and Steele, 2016), we and others have analysed and interpreted cancer data which suggest that DNA C-to-U deamination at off-target (i.e. non-Ig) genomic sites by AID/APOBEC deaminases triggers the recruitment of an ‘Ig SHM-like’ response (Lindley, 2013; Lindley and Steele, 2013). Many non-Ig protein-coding exons exist with (CWG)n repeat loci rich in clustered WGCW motifs (Willadsen et al., 2013). We also call these deamination events at off-target sites ‘dysregulated Ig SHM-like responses’ (Steele and Lindley, 2010; Lindley, 2013; Lindley and Steele, 2013; Lindley et al., 2016). We now know from the recent TCGA analyses of Niavarani et al. (2018) that TNR expansions are a very common feature in pan-cancer genome exomic data, accounting for 1–2% of DNA sequence modifications in cancer genomes, a similar frequency to the in-frame codon repeats observed in human Ig SHM data, as reported by Wilson et al. (1998a,b), and Reason and Zhou (2006). Thus, dysregulated in-frame expansions while rare are frequent enough outside the brain to be detected during SHM itself and in progressing somatic diseases such as cancer.

We propose that for an expansion event to be potentiated during complementary DNA (cDNA) synthesis, facilitated by DNA polymerase-η extension of the slipped MSH2–MSH3-stabilized CAG hairpin in the transcribed strand (TS) generated via reverse transcription (Figure 3), the pre-mRNA target site reverse transcription (TSRT) process (Luan et al., 1993) must allow the cDNA to invade the site (Box 1) to create new mutated heteroduplex DNA. As discussed in the legend to Figure 1 and Box 1, this ‘strand invasion’ process could be of limited tract length (≤30 nucleotides) by co-option of the 5′ and 3′ nicks on the TS by the complex responsible for the normal transcription-coupled repair (TCR) pathway of nucleotide excision repair (NER) known as NER-TCR (see Figure 2 in Hanawalt and Spivak, 2008; Spivak, 2016). This would create a ∼30 nt gap (Bowman et al., 1997) that can be filled in by DNA polymerase-η performing reverse transcription extending from the nicked 3′–OH–primed TS DNA. Alternatively, the 3′-OH TS priming site can be generated by AID-mediated deamination at the WGCW sites in the repeat (CAG)n or (CTG)n tracts. The length of such cDNA tracts synthesized by DNA polymerase-η can be promoted by the homologous recombination (gene conversion) properties that are also associated with its activity. In the chicken Ig gene conversion system, DNA polymerase-η deficiency causes a significant decrease in the frequency of gene conversion, with increased tract lengths in residual gene conversion events (Kawamoto et al., 2005). These results are compatible with other biochemical studies on homologous recombination promoting properties of human DNA polymerase-η (McIlwraith et al., 2005). As also addressed in Box 1, the results can be understood as DNA polymerase-η promoting DNA synthesis from strand invasion intermediates of homologous recombination, thus allowing invasion of the target V(D)J sequence by a pseudo V gene donor in the case of chicken gene conversion to generate a new Ig variable region-encoding tract (which can be up to several 100 nucleotides in length, in contrast to the ∼30 nucleotide gap synthesis tract in conventional NER-TCR just discussed). A D-loop is a DNA structure in which the duplex DNA is separated (unwound) and then held apart by a third homologous DNA strand in a triplex structure. In other triplex RNA-DNA structures, weaker non-Watson-Crick base pairs form Hoogsteen base pairing in which the third strand can be either in parallel or in the reverse orientation (see Li et al., 2016; Buske et al., 2012). In these structures, the triplex base hydrogen bonding involves non-Watson-Crick Hoogsteen hydrogen bonding such that A can pair with A, G with G and so on, pairings which are far weaker in strength but allow specific sequence identification over a longer region. An example of this type of triplex sequence matching can be found in Buske et al. (2011, 2012) for AG-rich enhancer/promoter regions.

## 5 Questions in advance

Given the expanded range of opportunities that we are now aware of for deaminases to target substrates in repeat tracts, it has been useful for us to pose the following three questions:

• What are the likely mechanisms of (CAGn)/polyQ and related TNR expansion diseases?
• Do these mechanisms incorporate all of the known molecular processes associated with expandable repeat diseases *in vivo*?
• How do repeat expansion diseases occur in the absence of cell division?

Answers to these questions are of special relevance to familial brain expansion diseases (Mirkin, 2007; Usdin et al., 2015; Richards, 2016; Polyzos and McMurray, 2017) and their idiopathic relatives (Bozza et al., 1995; Ishikawa et al., 1999; Kim et al., 2007).

Here, we propose an augmented molecular explanation that implicates the involvement of an alternate template and an alternate DNA repair polymerase (both of which occur in the absence of DNA replication) in known localized DNA-based repair mechanisms. Fundamental to our expanded view we ask: Is there a plausible role for pre-mRNA template intermediates and target-site reverse transcription involving DNA polymerase-η (as implicated for Ig SHM *per se* at rearranged V(D)J genes as shown in Figure 1) in TNR and related repeat expansion diseases occurring in transcribed regions of the genome (Figure 3)?

Another unanswered question is why such diseases are particularly prominent in brain and neuronal cells? For this, we have no specific answer. A general answer could involve the concept of the ‘inflamed brain’ and prion misfolding diseases as recognized now in Alzheimer's disease (Jaunmuktane et al., 2015; McCaulley and Grush, 2017). This concept is gathering momentum, both in scientific circles and in the clinic. It has much validity as it is known that chronic inflammatory diseases in the periphery, via immune cytokine cascades, can functionally communicate across the blood–brain barrier and activate enhanced “innate immunity states” in the microglial cells of the brain with untoward dysregulated consequences for normal brain function (Bullmore, 2018). Thus, tissue-localized aberrations in molecular innate immunity through off-target AID/APOBEC activation and targeting of C residues within deamination motifs (the prominent WGCW motifs in CAG repeats) might as a consequence precipitate aberrant Ig SHM responses in brain tissues. Disease triggers, in combination or in part, might include surgical central nervous system injuries associated with spinal taps (Gal-Mark et al., 2017), chronic peripheral inflammatory diseases (Bullmore, 2018), head knock brain injuries, pathogen infections and autoimmune inflammatory infiltrations in conjunction with a leaky blood–brain barrier (Myslinski, 2014; Montagne et al., 2015), or triggering of intrinsic endogenous dysregulated innate immune responses by snap-back dsRNA TNRs themselves (Richards et al., 2018; van Eyk et al., 2019). Activation of APOBEC and ADAR deaminases are known consequences of interferon-dependent innate immune response cascades (Schoggins and Rice, 2011; Schneider et al., 2014). In healthy brain tissues, expression of AID/APOBEC deaminases are low to undetectable (Refsland et al., 2010; Koning et al., 2009), while variable expression of ADAR isoforms are a normal physiological feature of the healthy brain (Picardi et al., 2015).

Finally, we ask, how do potentially expandable pathogenic TNR and related tracts arise in non-protein-coding introns as well as 5′ and 3’ untranslated regions (Mirkin, 2007; Usdin et al., 2015; Polyzos and McMurray, 2017)? One possibility is that they have been dispersed there in the evolutionary past as a consequence of retrotransposition events of fragments of RNA transcripts from coding regions. These transcribed yet non-protein-coding repeats often predispose to initiation of harmful non-ATG-mediated translated protein products, which can be very toxic to the cell (Pearson et al., 1997; Cleary et al., 2018).

Thus, the question again is: How are such (CAG)n and related repeats expanded in pre-mRNA in the absence of cell division? We propose that the error-prone RT mechanism for Ig SHM (Figures 1 and 2, Box 1, Box 2) has the potential to be adapted as an explanation for (CAG)n and related expansion diseases, thereby accounting for the generation of variant pre-mRNA expanded repeats (Figures 3 and 4), as well as potentially mutated sequences (i.e. somatic point mutations) in the flanking regions of TNR expansions. These new pre-mRNA sequences are then cDNA copied and locked back into the chromosomal DNA at that site (Luan et al., 1993) via the targeted RT action of DNA polymerase-η (or DNA polymerase-κ, Figure 4). These potential repeat expansion steps can also be adapted to the normal physiological Ig SHM process where about 1–2% of variant sequences in a hypermutated set of somatically mutated derivatives of rearranged V(D)J regions contain short 1–3 nucleotide indel repeats, including the previously discussed (Steele, 2016) in-frame codon expansions and contractions (Wilson et al., 1998a, 1998b; Reason and Zhou, 2006). Moreover, the proposed mechanism can also potentially lead to repeat contractions through hairpin removal by local DNA repair prior to reverse transcription. If these processes take place in the transcribed regions of expressed genes in germ cells, similar consequences for polymorphism generation are possible if off-target ‘Ig SHM-like’ responses are so activated.

We therefore expect the TNR expansion mechanism proposed here to be a general mechanism occurring across the genome for at least the (CAG)n, (CTG)n and (CGG)n diseases, as well as being a feature of other progressive somatic diseases such as cancer (see Dai and Wong, 2003 regarding breast cancer, and Niavarani et al., 2018 regarding many other cancers). Cancer genomes are known to display the mutation signature of AID/APOBEC off-target Ig SHM-like responses (Lindley, 2013; Lindley and Steele, 2013; Lindley et al., 2016). Further, the varied secondary RNA fold-back dsRNA structural conformations produced from TNR loci that are mutated are likely to be functionally altered, particularly via ADAR1-mediated RNA and DNA editing in RNA:DNA hybrids at transcription bubbles (Steele and Lindley, 2017) and also at A/C mismatches themselves in post-transcriptional snapback dsRNA structures in both exons and in 5′ and 3’ UTRs. Such A-to-I alterations could thus disrupt evolutionarily conserved RNA secondary structures in regulatory long non-coding RNAs (Smith et al., 2013, 2017).

## 6 What is the mechanism of CAG repeat expansions in non-dividing cells?

Most models of TNR expansion depend on hairpin-stabilized slippage and are based on DNA replication and repair models (Panigrahi et al., 2005; Mirkin, 2007; Chan et al., 2013 Guo et al., 2016), although local DNA repair models are being increasingly considered (reviewed in Polyzos and McMurray, 2017). Indeed, MSH2-associated mismatch repair deficiency actually leads to an absence of CAG repeat expansion, indicating that CAG repeat expansion *requires* an intact mismatch repair system (Manley et al., 1999). Hairpins in the leading strand, if stabilized (by MSH2–MSH3), can generate stable slippage events opposite the template DNA strand allowing priming of further leading strand DNA synthesis. After this, the relaxation of the retained hairpin leads to expansion of the number of TNR at the site of CAG repeats and other TNRs in the leading strand.

In our view, there are two possible molecular explanations for local DNA repair mechanisms based on stabilized hairpin slippage priming:

**Mechanism 1**. The first is based on DNA replication of the leading strand or localized DNA repair (Mirkin, 2007). This is a repair pathway involves DNA polymerase-β (assisted by DNA polymerase-δ) recruited by MSH2–MSH3 heterodimers targeting CAG repeat hairpins and resulting in stable slipped structures for priming of synthesis of the leading DNA strand (as described by Chan et al., 2013, Guo et al., 2016, and as extensively further reviewed by Polyzos and McMurray, 2017). This model can explain (CAG)n expansions in the DNA of non-dividing cells via conventional localized DNA repair concepts. We also note that, in yeast model systems of FRDA, RNA:DNA hybrid instabilities have been explained via transcription-generated RNA:DNA hybrids promoting (GAA)n repeat expansions in FRDA via break-induced DNA replication (Neil et al., 2018). Both of these mechanisms, however, can be classified as DNA-based.

**Mechanism 2**. The second possible explanation is based on mutagenic polynucleotide copying sequelae at transcription bubbles (Figure 1) now applied to CAG expansion diseases (Figures 3 and 4). This implies an error-prone RNA/RT-based DNA repair pathway, triggered and involving an ‘off-target Ig SHM-like response’. This invokes: **a.** specific codon-context targeting of cytosines by AID/APOBEC deaminases to unpaired C residues in ssDNA regions of both the displaced NTS and the TS during transcription (see Figure 1); **b.** translesion Y-family DNA polymerase-η synthesizing DNA opposite RNA (Franklin et al., 2004, Su Y et al., 2017, Su Y et al., 2019, Box 2), and; **c.** ADAR1 targeting at and around transcription bubbles via binding to negatively supercoiled Z-DNA (Steele et al., 2006). This RT model of Ig SHM has also been advanced to explain off-target (non-Ig) somatic mutagenesis across the progressing cancer genome (Steele and Lindley 2010, 2017; Lindley, 2013; Lindley and Steele, 2013; Lindley et al., 2016).

The off-target Ig SHM-like model, based on aberrant DNA repair and reverse transcription, is assumed to involve recruitment of DNA polymerase-η via MSH2–MSH3/MSH2–MSH6 heterodimers engaging G•U mispairs and short bulges/hairpins (compare Wilson et al., 2005). Shown in Figures 3 and 4, this model also provides a plausible explanation for such (CAG)n expansions in non-dividing cells, but also now involving the RT activity of an additional Y-family member, namely DNA polymerase-κ (Franklin et al., 2004). This could be triggered by an innate immune response to localized inflammation that activates off-target AID/APOBEC-mediated deamination of C residues in the canonical WGCW motifs in the NTS and the TS during transcription (Steele and Lindley, 2017).

The second explanation (Figures 3 and 4) is identical in every way to the first DNA-based model, except that the copying template is now the homologous sequence embodied in the pre-mRNA. Additionally, the DNA repair enzyme is the Y-family translesion DNA polymerase-η (and potentially DNA polymerase-κ as well), which performs reverse transcription to synthesize an error-prone cDNA copy of the TS, a downstream process after passage of the transcription bubble through that transcribed region. In our view, the second explanation provides a plausible sequelae of molecular steps to explain (CAG)n expansions at RNA polymerase II-transcribed regions in both dividing and non-dividing cells.

Is the signature of C-to-U deamination mediated by the AID/APOBEC enzymes evident in (CAG)n expansion data? In all (CAG)n expansion sequence collections examined, there are (CAG)n tracts interspersed with CAA codons (also coding for glutamine). So, these third position silent mutations do not change the protein sequence. However, as Nalavade et al. (2013) point out:

“Silent mutations in CAG repeats can also lead to disease, such as SCA2 (caused by CAG repeats in ataxin 2 [ATXN2]), wherein the CAA codons normally interspersed within the CAG repeat are absent in patients, leading to an enhanced uninterrupted CAG repeat. As CAG and CAA both code for Q, there are no resulting amino-acid changes, indicating that mRNA level changes are sufficient for disease development. There are significant structural consequences of CAA interruptions on the hairpin formations...”

We quote this in full because it touches on key aspects of our expanded molecular explanation. Also, it underlines the fact that ‘toxic’ RNA structures can be the result of TRN expansions in transcribed regions. We expect C-to-U deamination signatures on both the NTS and the TS at transcription bubbles as well (Figure 1). A direct C-to-U change in the NTS at the first position of the codon would result in the creation of ‘TAG’ stop codons thus resulting in N-terminal truncated proteins which are likely to never be recovered in DNA sequence collections (owing to purifying selection and thus censorship through nonsense-mediated decay and apoptotic cell deletion). But a G-to-A change in the third position is common. This strongly suggests C-to-U editing of the complementary DNA strand (i.e. the TS), resulting in a G-to-A mutation being incorporated into the synthesized pre-mRNA strand (as shown in Figure 1). If this deamination event is the result of an AID-mediated deamination, then it may also activate further off-target Ig SHM-like responses at polyCAG and similar tracts.

Some further information and references supporting the RNA/RT mechanism proposed here are included in Figures 1, 2, 3, and 4, as well as Boxes 1 and 2. The entire spectrum of somatic point mutations known to be associated with off-target Ig SHM-like activity occur at and around the initial uracil lesion that results from C-to-U editing in DNA (Figure 2). With respect to the NTS (by convention), *in vivo* data also show that mutations at A residues exceed mutations at T residues (A >> T) and mutations at G residues exceed mutations at C residues (G >> C) as expected by the RNA/RT mechanism given that the great majority of base substitutions and base modifications accrue in the pre-mRNA, as highlighted in Figure 1 and discussed in Figure 2 legend (Steele, 2009; Lindley and Steele, 2013). This is expanded on in the Supplementary Information File.

The deaminase-driven RT off-target Ig SHM model outlined here is consistent with the recent data reported by Su and Freudenreich (2017). These investigators showed that a dependence on C residue deamination-coupled BER resulted in CAG repeat fragility and instability in *Saccharomyces cerevisiae*. It is also consistent with the data reported in Neil et al. (2018) on RNA:DNA hybrid instabilities promoting GAA repeat expansions in FRDA via break-induced DNA replication. These reports are also consistent with our proposal that CAG and GAA repeat fragility and expansion data fit within the aegis of the RT Ig SHM model, albeit now acting in a dysregulated and off-target manner across the genome and guided by pre-mRNA templates around which all the mutagenic action depends (Figures 1, 3, and 4).

## 7 Direct evidence for DNA polymerase-η involvement in (CAG)n expansions?

Reviewers have made us aware of the work of Dixon and Lahue (2002) in a yeast model system. (CAG)n contraction and expansion in this system is essentially unaffected by a deficiency in DNA polymerase-η (Rad30), suggesting that it has little or no effect on either CAG contraction or expansion. We note, however, that one of the three contraction trials reported by Dixon and Lahue (2002) showed a five-fold reduction in (CAG)n contractions in the absence of DNA polymerase-η (while no effects on expansions were noted in other experiments). Several points can be made about these data. Yeast model systems emphasize replicative (CAG)n expansions (or contractions). A number of polymerases acting in concert with MSH2–MSH3 heterodimers could be involved. Apart from the two investigated yeast translesion DNA polymerases-η and -ζ as such (and thus potentially assisting (CAG)n expansions/contractions), there is the potential RNA-templated DNA repair activity of yeast DNA polymerases-α and -δ (Storici et al., 2007). It is therefore not surprising to us that there is little effect of DNA polymerase-η ablation in such a system. Our focus here is exclusively on RNA polymerase II-transcribed regions in non-dividing human cells (the situation that would be expected to apply in the brain). In these type of situations, off-target or dysregulated AID/APOBEC (and ADAR) deaminases can potentially target C residues (and A residues) in substrates in the context of stalled transcription bubbles at RNA:DNA hybrids (Steele and Lindley, 2017). In a normal length 10–30 nucleotide CAG repeat tract (Usdin et al., 2015; Richards, 2016) we can expect that the tandem array of AID motifs (i.e. WGCW) will present many potential deamination targets. Many WGCW motifs could be deaminated in a dysregulated ‘hypermutation’ episode inducing a DNA damage response to the affected CAG repeat tract. Indeed, prior DNA duplex damage by reactive oxygen species and 8oxoG generation can also be expected as a common DNA damage event at (CNG)n tracts (Polyzos and McMurray, 2017, Figure 4). If an innate immune response initiates a deaminase-driven ‘hypermutation’ cascade, this could potentially mutate several of the numerous WGCW tandem sites in the normal 10–30 nucleotide repeat (AID/APOBEC enzymes tend to act in a processive manner on DNA substrates, Chelico et al., 2009), and the RNA polymerase II complex will certainly sense this type of DNA damage in advance and organize to backtrack and recruit the NER-TCR repair machinery (Hanawalt and Spivak, 2008; Spivak, 2016). The density of WGCW motifs in CAG repeat tracts that could act as deamination targets of AID is similar to the high concentrations of the same or similar AID motifs in rearranged V(D)J genes and the proximal intergenic regions (see these features in the *in vivo* hypermutated VκOxJκ5 transgene sequence described in Steele et al., 2006 and the VκOxJκ5 and downstream Jκ5-Cκ sequence presented in the supplementary data of Steele, 2016).

## 8 Frequency of such RT-driven expansion events *in vivo*?

A reviewer has also proposed that the frequency of the proposed RT expansion events (Figures 3 and 4) is likely to be low, compared to the frequency of TNR expansions observed in model systems where instability thresholds are reached very frequently. Many model systems would be of the ‘replicative-type’ so the issues surrounding non-dividing cells would not necessarily apply. That said, we agree with this expectation. Furthermore, we anticipate that these hypothesized events (detailed in Figures 3 and 4 as per Figure 1), like the neuronal diseases themselves, are usually rare. But in the affected non-dividing cells of the brain they could have significant impact. Untoward ‘inflammatory’ triggering events with deaminase activities targeting WGCW motifs could be important to pathology. As discussed above, these could be external through breaches in the blood–brain barrier, or endogenous to affected brain cells related to ‘anti-self innate immune responses’ to the snap back dsRNA repeat hairpins themselves and involving ADAR1 (Liddicoat et al., 2015; Richards et al., 2018; Samuel, 2019; van Eyk et al., 2019).

## 9 Summary

**Figure 5:**
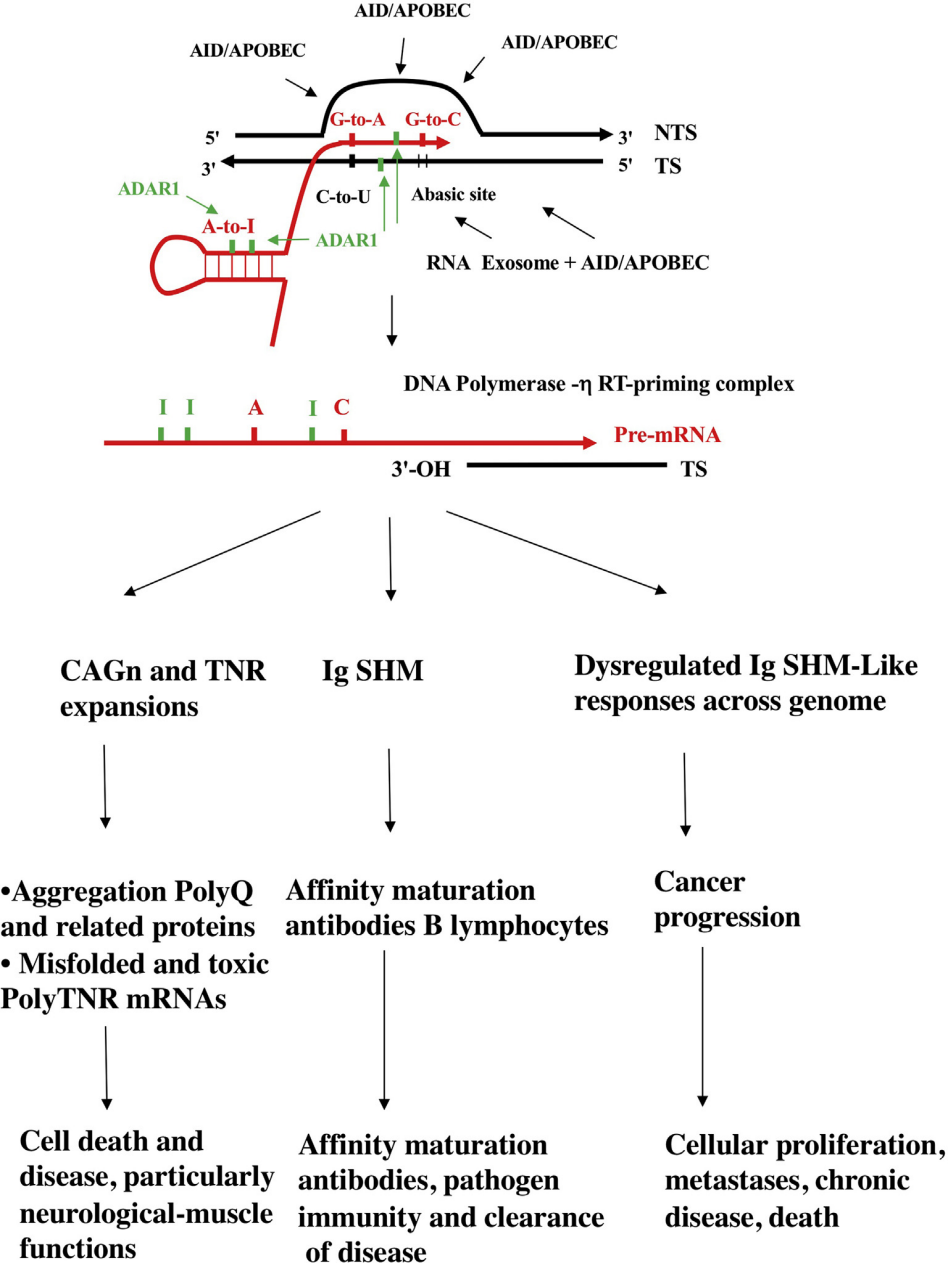
Deaminase and reverse transcriptase-driven mutagenesis at transcription bubbles.

Our main deduction is that the RT model proposed for (CAG)n and similar expandable repeats draws upon the implications of what we now know about off-target Ig SHM-like mechanisms. The steps for RNA/RT (CAG)n repeat expansions shown in Figures 3 and 4 are thus plausible within the context of a RNA/RT model involving off-target deaminase-driven Ig SHM-like responses (Figure 1). Figure 5 summarizes this model with respect to the downstream sequelae of deaminase-mediated and RT-coupled mutagenesis at transcription bubbles. When this process is confined to Ig loci in the regulated environment of the hypermutating germinal center B lymphocyte, the beneficial result to immunological health is the affinity maturation of antibodies and protection against disease. However, it is clear that when dysregulated there is very great potential for somatic mutagenesis at multiple off-target genomic sites causing serious pathologies, such as suggested here for repeat expansion diseases in non-dividing cells and for cancer progression as described elsewhere.

Thus, we propose here a distinct set of additional molecular concepts to provide a significantly augmented way of viewing the molecular mechanism of repeat expansion diseases, particularly (CAG)n-based diseases, appearing in the protein-coding regions of genes expressed in non-dividing cells. This view combines new additional pathways with pre-existing thought on (CAG)n repeat generation processes. The main advantages of adopting a RNA/RT-based model are:

1. It will always expand the CAG repeat or related TNR tract in the 5′-to-3′ direction as is routinely observed (although mismatch DNA repair-mediated contractions are also predicted to take place).
2. The main repeat motifs, AGCA or TGCT, conform to the known prominent AID-targeting deaminase motif (i.e. WGCW), which when deaminated (C-to-U) is known to activate locus-specific Ig SHM and class switch recombination. The existence of the interspersed CAA repeat in (CAG)n tracts is consistent with C-to-U deamination of the C-site on the complementary strand in the evolutionary past; that is, there is, in our view, a clear evolutionary remnant of the AID deamination C-to-U signature on the TS (the template for pre-mRNA synthesis) that is found in all data sets (the absence of the TAG signature is explained by purifying selection).
3. The RT activity of human Y-family DNA polymerases-η and -κ, given that both are *bona fide* high-rate error-prone translesion repair polymerases, feasibly contributes to both somatic mutation during disease progression as well as germline polymorphism in the CAG and related TNR repeats and surrounding sequence.

## Declarations

### Author contribution statement

All authors listed have significantly contributed to the development and the writing of this article.

### Funding statement

This research did not receive any specific grant from funding agencies in the public, commercial, or not-for-profit sectors.

### Competing interest statement

The authors declare no conflict of interest.

### Additional information

No additional information is available for this paper.

## Appendix A Supplementary data

Supp Info File Supp Info File
