# Supplementary material for: A proposed reverse transcription mechanism for (CAG)n and similar expandable repeats that cause neurological and other diseases
Source: Heliyon. 2020 Feb 26;6(2):e03258. doi: 10.1016/j.heliyon.2020.e03258 (PMC7044655; doi:10.1016/j.heliyon.2020.e03258)
Supplement: Supp Info File [file mmc1.pdf]

Supplementary Information File

To : A. Franklin, E.J. Steele, R.A. Lindley. (2020) A proposed reverse transcription mechanism for (CAG)n and similar expandable repeats that cause neurological and other diseases. Published in 2020 in Heliyon. The references are the same as in the main version of the paper except for additional ones added here.

The published evidence suggests that AID targets both the NTS as well as the TS in ssDNA in the context of the 'open' transcription bubble as shown in Figures 1 (figure reproduced again here, not the legend). Additionally, the work of Basu et al (2011) characterizes the important role of the RNA exosome in allowing AID deaminase access to non-paired cytosines for C-to-U deamination on the TS in the context of the RNA:DNA hybrid at the transcription bubble (Figures 2 in the main paper).

Figure 1. Reverse Transcriptase Mechanism of Ig Somatic Hypermutation

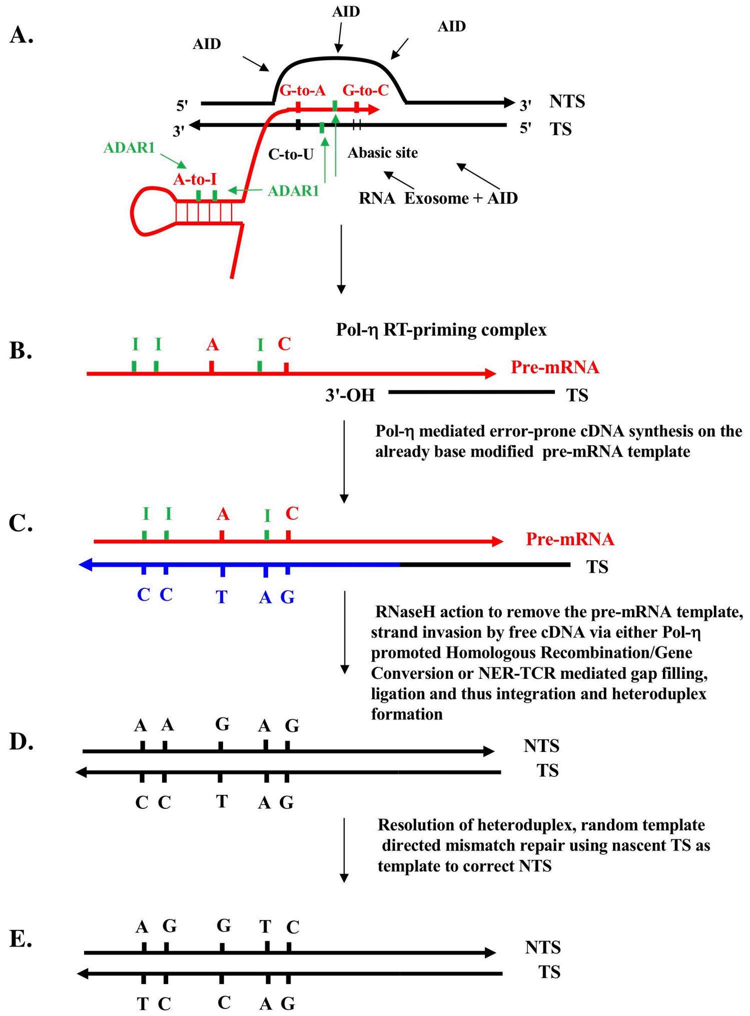

The RNA/RT model of Ig SHM explains strand-biased mutations at both A:T and G:C base pairs , where mutations of A exceed mutations of T (A>>T) and mutations of G exceed mutations of C (G>>C, Steele et al 2004, Steele et al 2004, Steele et al 2006, Steele 2009). The key to these counter intuitive strand biased patterns (e.g. see Lindley and Steele 2013) is the involvement of DNA polymerase-η mediating RNA-directed error-prone cDNA synthesis (Franklin et al 2004) copying the already base-modified Ig pre-mRNA template. The RNA base modifications can occur either via A-to-I editing at W<u>A</u>-sites in nascent dsRNA stems emergent from the transcription bubble (Steele et al 2006), the A-to-I editing of RNA and DNA

moieties at WA-sites in RNA:DNA hybrids (Steele and Lindley 2017), or nucleotide misincorporation by RNA polymerase II opposite AID-generated U residues and subsequent Uracil-DNA glycosylase-generated abasic sites in template DNA (Kuraoka et al 2003) on the TS (as shown in Figures 1, 2).

The model thus predicts the annealing and formation of a RT priming complex between the 3'-OH terminal of the nicked TS that results from processing of an AID-induced lesion (at WGCW and related AID-motifs such as WRCG) and the Ig V(D)J pre-mRNA (Figure 1B), followed by cDNA is synthesis towards the transcription start site (Figure 1C). As shown in steps Figure 1D and 1E, this leads to the integration of now error-filled cDNA copies back into the normal chromosomal site by the TSRT mechanism (Luan et al 1993). Thus, the modern form of the RNA/RT mechanism (Franklin et al 2004, Steele 2016, Steele and Lindley 2017) depends on AID-generated C-to-U lesions, a base modified pre-mRNA copying template and then long tract error-prone cDNA synthesis (Blanden et al 2004) opposite this pre-mRNA template by DNA polymerase-η to produce a new variant copy of the TS (see further explanations in the legend to Figure 1 and in Boxes 1 and 2). It is actually a form of TCR (Hanawalt and Spivak 2008, Spivak 2016), but now involving target site directed integration of cDNA tracts into the genome so as to replace that section of the previously damaged TS (see the legends to Figures 1 and 4). This is a form of RNA template-mediated repair as previously proposed by Trott and Porter (2006) and shown in yeast by Storici et al (2007).

In Figure 1, this integrated mutagenesis processes can be understood in more detail as per the following steps:

- As the pre-mRNA emerges from the transcription bubble, snapback nascent stem loops form as shown (and see more exact details in Steele et al 2006). These present readily accessible WA sites on the proximal 3' side of the dsRNA stem to the ADAR1 deaminase which is complexed via Z-DNA binding sites to the elongating RNA polymerase II complex allowing immediate A-to-I editing of the emergent dsRNA substrates in the pre-mRNA prior to splicing (Herbert et al 1997, Herbert and Rich 2001).

- As the transcription bubble passes through the region, the emerging polyribonucleotide becomes a base-modified pre-mRNA, and is then free to invade the chromosomal duplex and form RT priming complexes with the nicked TS, with 3'-OH ends as shown in Figure 1B. The single-stranded prime site nicks are thought to result from AID lesions (but can also be plausibly generated by NER-TCR processes as discussed in the legends to Figures 1 and 4). As the cDNA is made (blue lines), the potential exists for both accurate and inaccurate DNA polymerase-η-mediated cDNA synthesis.

- Further error-prone events are predicted as DNA polymerase-η may miscopy opposite I residues in the pre-mRNA (not shown).

- At RNA:DNA hybrids, both DNA and RNA moieties can be A-to-I edited (Zheng et al 2017) adding further to the strand-biased spectrum at A residues with respect to the NTS, but the T-to-C here results from direct A-to-I editing of the DNA of the TS in the RNA:DNA hybrid (Steele and Lindley 2017).

- There are further possible downstream editing events involving base exchanges at mismatches (Figure 1E) to resolve the heteroduplex before replication occurs (McPhee 1995).

- 8oxoG modifications of the pre-mRNA are not shown in Figure 1 but may further contribute to the strand bias of G-to-T > C-to-A transversions in cancer genomes as discussed (Steele and Lindley 2010, Lindley and Steele 2013). However 8oxoG modifications of G residues are prominent in the DNA of CAG repeat tracts (Polyzos and McMurray 2017) and this is highlighted in Figure 4 as a NER-TCR triggering lesion.

• Finally it needs to be mentioned that the group of I. G. Rogozin and colleagues showed 20 years ago that WA-sites are also the preferred motif for DNA polymerase eta and is cited in our key papers e.g. Franklin et al 2004, Steele et al 2004, Steele et al 2006, Steele 2009, Steele 2016 etc. This fact is common knowledge in the SHM field. Two primary references we cited in our earlier papers are Rogozin et al 2001, Pavlov et al 2002 (as have many others in the Ig SHM field, Di Noia and Neuberger 2007, Teng and Papavasiliou 2007, Maul and Gearhart 2010).

Using our proposed step-wise flow, the model described in Figure 1 can potentially result in the full spectrum of strand biased somatic mutations at A exceeding mutations at T (A>>T) and somatic mutations at G exceeding mutations at C (G>>C) as known to be generated *in vivo* during Ig SHM (Steele 2009, Steele and Lindley 2017) and in TP53 substrate sequences in cancer genomes (Lindley and Steele 2013, Steele and Lindley 2017). Thus our analyses suggest the same processes occur in cancer genomes during off-target Ig SHM-like responses (Lindley 2013, Lindley and Steele 2013, Lindley et al 2016), accentuated most likely by combinatorial multimerization of AID/APOBEC deaminase binding domains during cancer progression (Mamrot et al 2019).

Additional References not in main paper

A. Herbert, A. Rich. The role of binding domains for dsRNA and Z-DNA in the in vivo editing of minimal substrates by ADAR1. Proc. Natl. Acad. Sci. U. S. A. 98 (2001) 12132–12137. DOI: 10.1073/pnas.211419898

A. Herbert, J. Alfken, Y.-G. Kim, I.S. Mian, K. Nishikura, A. Rich. A Z-binding domain present in the human editing enzyme: double-stranded RNA adenosine deaminase. Proc. Natl. Acad. Sci. U. S. A. 94 (1997) 8421–8426. DOI:10.1073/pnas.94.16.8421

Y.I. Pavlov, I.B. Rogozin, A.P. Galkin, A.V. Aksenova, F. Hanaoka, C. Rada, T.A. Kunkel. Correlation of somatic hypermutation specificity and A-T base pair substitution errors by DNA polymerase η during copying of a mouse immunoglobulin k light chain transgene, Proc. Natl. Acad. Sci. U.S.A. 99 (2002) 9954–9959.

I.B. Rogozin, Y.I. Pavlov, K. Bebenek, T. Matsuda, T.A. Kunkel. Somatic mutation hotspots correlate with DNA polymerase η error spectrum. Nat. Immunol 2(2001) 530–536
